# Changes in spontaneous movement in response to silent gaps are not robust enough to indicate the perception of tinnitus in mice

**DOI:** 10.1371/journal.pone.0202882

**Published:** 2018-08-29

**Authors:** Daniël O. J. Reijntjes, Nick M. A. Schubert, Alexander Pietrus-Rajman, Pim van Dijk, Sonja J. Pyott

**Affiliations:** University of Groningen, University Medical Center Groningen, Department of Otorhinolaryngology and Head/Neck surgery, Groningen, The Netherlands; University of Otago, NEW ZEALAND

## Abstract

Approaches to identify the perception of tinnitus in various animal models have been difficult to apply to mouse. As a result, mice have been underutilized to investigate the cellular, molecular, and genetic mechanisms underlying tinnitus. A recent study in guinea pigs identified a novel spontaneous behavior (unconditioned response), changes in movement during silent gaps, that identified a subgroup of animals presumably with tinnitus. Guinea pigs identified with tinnitus failed to “freeze” in response to silent gaps in sound. In the hope of developing a rapid and reliable assay for mice, we used a similar approach. C57BL/6J mice underwent three trials in which spontaneous movement was video recorded in the presence of white noise interrupted with six silent gaps. Movement metrics included velocity and body movement. Before the third trial, mice underwent either sham or noise exposure to induce hearing loss and tinnitus. Auditory brainstem responses before and after noise trauma confirmed normal hearing in sham-treated animals and hearing loss in the noise-exposed cohort. No differences in the various movement metrics were detected during the silent gaps either before or after sham/noise exposure. Variability in spontaneous movement both before and after sham/noise exposure was substantially greater in mice compared to guinea pigs. Thus, this assay is not sufficiently statistically powerful to identify changes in movement that might indicate tinnitus perception in mice. Previous observations also reported increased movement overall in guinea pigs identified as suffering tinnitus. In contrast, mice showed no statistically significant differences in movement between the three trials. Despite our results, other unconditioned (as well as conditioned) behaviors should be examined in mice to test their utility to detect changes that indicate the perception of tinnitus. Such assays are essential to accelerate the use of mouse models in tinnitus research.

## Introduction

Subjective tinnitus is the phantom perception of sound without a measurable stimulus. The prevalence of tinnitus increases with age and affects an estimated 5 to 40% of the adult population [1]. Despite the high prevalence and also enormous personal and societal burden of tinnitus [2,3], existing treatment options are largely limited to coping strategies [4]. Limited understanding of the pathophysiological mechanisms underlying tinnitus perception is one of the main reasons for the lack of effective treatment options. Research into the cellular, molecular, and genetic mechanisms underlying tinnitus requires animal models that permit the detection of tinnitus perception. Existing animal models utilize either spontaneous or conditioned responses that are difficult to apply in mouse, have low throughput, and/or have questionable relevance to human tinnitus perception. As a result, the experimental advantages of mouse models have been underutilized to investigate the pathophysiology underlying tinnitus.

Current animal models to detect the perception of tinnitus utilize one of two main approaches: 1) conditioned responses based on work by Jastreboff and colleagues [5] or 2) gap-prepulse inhibition to acoustic startle (GPIAS) based on the work by Turner and colleagues [6]. Conditioning responses require a learned (conditioned) response, often to detect silent gaps that are presumably masked by the presence of tinnitus. Various conditioned responses have been investigated, including conditioned avoidance and positive reinforcement paradigms [7]. Mice are rarely used to examine conditioned responses because mice are considered less amenable to training [7]. GPIAS has become very popular among researchers for its speed and ease of use and has been implemented in many mouse studies [8]. GPIAS relies on the acoustic startle response following a brief, loud sound. The magnitude of the acoustic startle is reduced when a silent gap in the preceding background noise is applied. In animals with presumed tinnitus, this reduction in muscle contraction is decreased, consistent with the masking of the silent gap by the perception of tinnitus [6]. Even though GPIAS use is widespread and several papers support the idea that GPIAS faithfully detects tinnitus [9,10], issues have been raised due to the reflex-like nature of the test, discrepancies in the tinnitus frequency area, and drops in baseline responses after noise exposure [8]. These issues have resulted in recent re-evaluations of GPIAS as a suitable model for the perception of tinnitus [11]. Complicating this paradigm, mouse strains show variability in their GPIAS responses [12]. In summary, currently available approaches to identify the perception of tinnitus have limited applicability to mice, and subtle experimental improvements on these models [12] do not necessarily overcome inherent limitations in these models. Therefore, different approaches are urgently needed for the detection of perception of tinnitus in mice. Ideally, these new approaches would be rapidly and reliably incorporated into high throughput hearing assessment pipelines.

A recent study identified a novel spontaneous behavior (unconditioned response), changes in movement during silent gaps, that appears to indicate the perception of tinnitus in guinea pigs [13]. Specifically, guinea pigs identified as perceiving tinnitus after unilateral noise exposure failed to “freeze” in response to silent gaps. As in other gap detection paradigms, the perception of tinnitus was expected to “fill” this silent gap and cause a loss in this freezing response. This response was quite dramatic, and the guinea pigs identified as perceiving tinnitus showed an approximate 30% reduction in “freezing” during silent gaps compared to either normally hearing guinea pigs or guinea pigs with noise-induced hearing loss that were not identified as perceiving tinnitus. Importantly, this response was unconditioned and required no training. Also, interestingly, this subgroup of guinea pigs identified as perceiving tinnitus was more active in general compared to either normally hearing guinea pigs or guinea pigs with noise-induced hearing loss but without tinnitus.

In this study, we modified this assay to test the utility of this spontaneous behavior (unconditioned response) to detect the perception of tinnitus in mice. We hoped to develop a rapid and reliable assay that does not require training or conditioned responses to identify the perception of tinnitus in mice. Moreover, we aimed to develop an assay that was not strain specific and, because most genetically modified mice are backcrossed onto the C57BL/6J strain, we chose to develop this assay in this strain of mice. Based on these previous findings in guinea pig, we utilized a modified experimental approach to test three specific predictions in mice:

1. Normally hearing mice will show quantifiable changes in movement (likely “freezing”) in response to silent gaps.
2. Mice exposed to noise consistent with the production of tinnitus will show a quantifiable loss of this change of movement (likely loss of “freezing”) in response to silent gaps.
3. Mice exposed to noise consistent with the production of tinnitus will show an increase in total activity compared no normally hearing or sham exposed mice.

Although we predicted that normally hearing mice would “freeze” in response to silent gaps and that noise exposed mice would show loss of “freezing” in response to silent gaps, we did not exclude the possibility that noise exposure expected to induce tinnitus might cause other changes in movement in response to silent gaps. Therefore, movement was assessed both before and after noise exposure, using the minimal number of mice required based on the expected effect size (see Methods), to identify any changes in spontaneous movement that might indicate the perception of tinnitus. Despite our predictions, we found that this assay was not sufficiently statistically powerful to identify changes in movement in mice that might indicate hearing loss and/or tinnitus perception in mice. Given the importance of identifying the perception of tinnitus in mice, other spontaneous, unconditioned behaviors as well as conditioned behaviors should be examined in mice to test their utility to indicate the perception of tinnitus.

## Materials and methods

### Animals

All experiments were approved by the animal ethics committee of the University of Groningen and University Medical Center Groningen and complied with all guidelines for animal experiments from the UG/UMCG and Netherlands animal welfare law. A total of 8 C57BL/6J male mice aged 6 weeks (at the onset of experiments) were used in this study and obtained from the stock maintained at the UMCG Central Animal Facility. The C57BL/6J substrain was confirmed via single nucleotide polymorphism (SNP)-based genome scanning (performed by Jackson Laboratories). 100% of the 150 SNP markers evenly spaced over the 19 autosomes and the X chromosome were identical in the C57BL/6J colony maintained at the UMCG Central Animal Facility compared to the substrain maintained by Jackson Laboratories (data not shown). The minimum number of experimental animals needed in these experiments (n = 3 mice per experimental group) was statistically calculated using the effect size measured in previous experiments with guinea pig [13]. In these experiments, repeated measures ANOVA with noised guinea pigs (n = 10) and sham noised guinea pigs (n = 5) yielded an F-statistic of 164.7. From this F statistic, the expected effect size (Cohen’s d) is 7.55. Setting the significance level (α) at 0.05 (two-tailed) and the power (1- β) at 0.95, the number of animals required per experimental group is 3. An additional animal per group was included to offset possible (but unlikely) animal attrition during the study duration. Noise exposure, expected to induce hearing loss and tinnitus, was performed under ketamine and dexmedetomidine anesthesia to minimize suffering. During the experimental periods, animals were housed in normally sized mouse cages. Cages contained a maximum of 3 mice. Food and water were provided ad libitum. Mice were observed daily for overall welfare. Body mass was monitored weekly and then daily following noise exposure and auditory brainstem response (ABR) measurements. After noising and ABR measurements, mice were allowed to recover in a monitored recovery room for 24 h.

### Auditory brainstem responses

Mice were anesthetized with an intraperitoneal injection of 75 mg/kg ketamine and 1 mg/kg dexmedetomidine and placed in an acoustic chamber. Electrode placement was according to Ingham et al. [14]. Both click and pure tone (8, 16, 32 kHz) ABRs were recorded from the left ear in response to sounds produced with an open field speaker (Visaton DHT 8 S) connected to an amplifier (Dynavox ET-100) and generated by dedicated hardware and software (Intelligent Hearing Systems). Stimuli had durations of 25 μs (clicks) and 5000 μs (tone bursts) and were played at a rate of 21.1 times/s. Sound intensities were calibrated at a marked distance of 3 cm from the speaker using a Brüel & Kjær piston phone (Type 4220), a Brüel & Kjær measuring amplifier (Type 2610), a Hameg instruments oscilloscope (HM303-6), and a Brüel & Kjær preamplifier (Type 2670) with ¼ inch Brüel & Kjær microphone (Type 4938). dB SPL was determined using peak to peak calibration as described previously [15]. ABRs were recorded starting at an intensity of 10 dB and increased to 90 dB with 5 dB increments. Electrode recordings were amplified 100× with bandpass filter settings of 30–3000 Hz. Responses were averaged over 512 recordings. To reduce the total time mice were under anaesthesia for ABR measurements, only responses from the left ear were recorded. Because noise exposure was free field, both ears were expected to be equivalently affected. Absolute thresholds were set at the first intensity where peak I was discernible above the noise floor and consistently present thereafter. Thresholds were marked independently by two observers and finalized after comparison. Threshold shifts were calculated by subtracting noise/sham exposure absolute thresholds from individual baseline absolute thresholds.

### Noise exposure

Mice were anesthetized with an intraperitoneal injection of 75 mg/kg ketamine and 1 mg/kg dexmedetomidine. Sham exposed mice were put in an acoustic chamber without presentation of noise. Experimental animals were placed in an acoustic chamber and exposed to a 10 kHz tone at a 120 dB SPL for 90 min using a free field speaker (Visaton DHT 8 S) coupled to an amplifier (Dynavox ET-100). The tone was generated using dedicated ABR hardware and software (Intelligent Hearing Systems). This paradigm was based on previously used paradigms to induce tinnitus in mice. For example, tinnitus has been induced in CBA/CaJ anesthetized with ketamine/xylazine and exposed to noise centered at 16 kHz (116 dB SPL) for 1 h [9]. In awake CBA/J mice, tinnitus has been induced by exposure to noise centered at 10 kHz (120 dB SPL) for 1 h [16]. Finally, in C57BL/6J mice tinnitus was induced by exposure to noise centered at 16 kHz (116 dB SPL) for 1 h following anesthesia with isoflurane [17].

### Movement monitoring in response to silent gaps

The behavioral assay consisted of an open field test in a custom-built arena (measuring 50 cm^3^). A speaker (Horn tweeter FT17H, Fostex Co.) and a camera (Nokia N8, Symbian Belle OS) were suspended above the center of the open field to provide the background sound stimulus and record the mouse’s movement in the open field. Mice were given a 1 min habituation period before the onset of the background sound stimulus. The background stimulus consisted of a 510 s white noise randomly interspersed with six silent gaps either 60, 75, or 90 s apart. Gaps were either 500 ms or 1 s in duration with 20 ms on and off ramps. Sound files were generated using Adobe audition CS6. The sound was calibrated to be 80 dB SPL at the floor of the open field arena with a negligible difference in dB SPL measured between the center and the corners of the arena. 80 dB SPL was chosen because this sound intensity is audible above the threshold shifts observed after noise exposure. In pilot experiments we also tested 60 dB but there were no discernible differences in mouse behavior or movement. For each trial different sound files (with differently interspersed silent gaps) were used. To promote activity, mice were put on a reversed 12 h:12 h light:dark cycle, and movement monitoring was assessed under red lights in the beginning of their dark (active) phase. Because mice show substantially more movement (and exploratory behavior) than guinea pigs, the duration of silent gaps tested in mice (0.5 and 1s) were shorter than the duration of silent gaps examined originally in guinea pigs (30, 60, 90, and 120 s) [13]. These durations were based on pilot results examining various shorter (0.5, 1 and 5 s) and comparable longer duration silent gaps. No differences were detectable in responses to silent gaps of either 0.5 or 1 s.

### Quantification of movement metrics

Video files were recorded at 30 frames per second in VGA resolution. Behavior was assessed by inspection of the video footage and quantified using Ethovision XT 11.2 [18]. Movement was quantified in two ways: 1) body movement or movement of all three body markers, which included head, tail, and torso (center point) and 2) velocity (center point). Values for both metrics were calculated 30 times/s using Ethovision XT 11.2 [18] and exported to Excel (Microsoft). The body movement metric was scored in a binary fashion: a score of 1 was given whenever at least one of the three markers was moving and a score of 0 was given when there was no movement of any of these three markers. These scores were converted into a percentage of activity over time using the following formula:

$$\left(\frac{events_{1}-events_{2}}{time\left(s\right)\times 30\;events/s}\right)\times 100.$$

For the second metric, velocity was averaged over time. These metrics were computed for each individual mouse for all three trials for 65 s surrounding each individual silent gap, including the 30 s preceding the onset of the gap, the 5 s containing the gap (that is, 0.5 or 1 s during the gap and an additional 4.5 s or 4 s after the gap), and the subsequent 30 s. To quantify the relative change in movement in response to the silent gap, data from 30 seconds preceding the onset of the gap was compared to data from 5 seconds after the gap for both metrics using the following formula:

$$\frac{Movement_{30s,pre}-Movement_{5\;s,gap}}{Movement_{30s,pre}}.$$

In this way, comparisons between changes in movement could be made even if absolute movement was very different across mice, gaps, and trials.

### Statistics

All values are presented as ± SEM. N refers to the number of animals. Data were not assumed to be normally distributed and, therefore, only nonparametric statistical analyses were used. The Mann Whitney rank comparison test was used for paired comparisons. The Kruskal Wallis test with Dunn’s multiple comparisons test was used for multiple comparisons. The Friedman test with Dunn’s multiple comparisons test was used for multiple comparisons of matched/repeated measures. The statistical analyses were performed in GraphPad Prism and are described in detail in the text. In all cases, P values ≤ 0.05 were used to establish significance.

## Results

### Experimental overview

**Fig 1:**
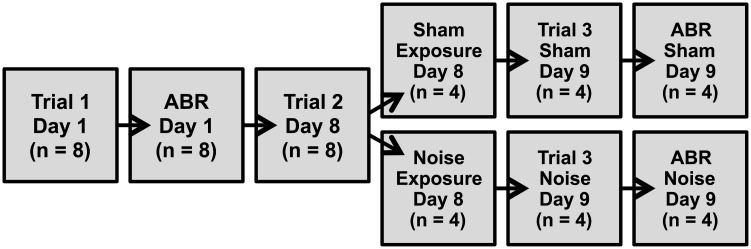
Experimental overview. Spontaneous movement was monitored in a total of three trials over 9 days. ABRs were performed (Fig 2) to verify normal baseline hearing (on day 1) and assess hearing loss after sham/noise exposure (on day 9). The first and second trials were performed (on days 1 and 8) to assess inter-test reliability of movement monitoring (Fig 3). The third trial was performed (on day 9) to assess changes in spontaneous movement following sham/noise exposure (Fig 4).

In these experiments, mice underwent three trials of movement monitoring (Fig 1). The first trial occurred on day 1 and was followed immediately by ABR measurements to assess baseline hearing. The second trial occurred on day 8 and was immediately followed by either sham or noise exposure. Mice were randomly allocated to either the sham or noise exposed groups. The third trial occurred on day 9 and was followed immediately by ABR measurements to assess threshold shifts due to sham/noise exposure. Thus, there were a total of four trial conditions: trial 1 (before sham/noise exposure), trial 2 (before sham/noise exposure), trial 3 (after sham exposure), and trial 3 (after noise exposure). The first two trials (before sham/noise exposure) provided an indication of the inter-test reliability of movement monitoring to detect changes in movement in response to silent gaps. This reliability is necessary since this paradigm requires a minimum of two trials (before and after sham/noise exposure) to assess changes in movement due to the perception of tinnitus.

### Auditory brainstem response (ABR) measurements to assess noise-induced hearing loss

**Fig 2:**
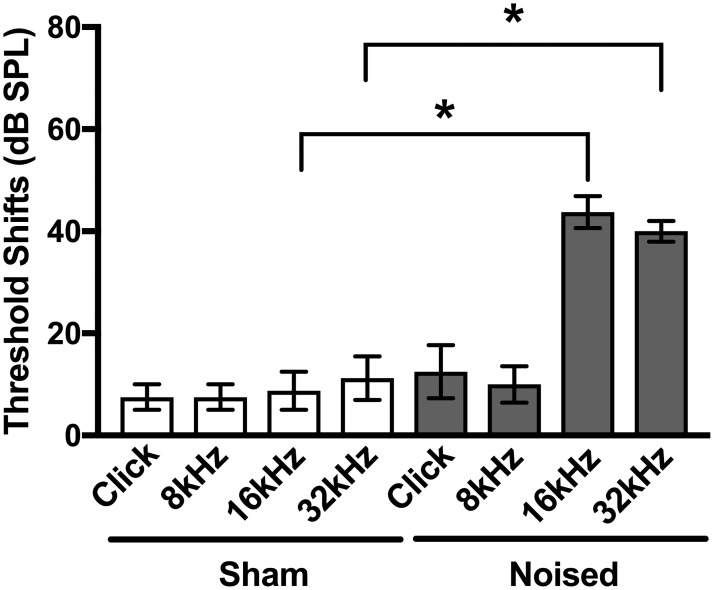
Absolute threshold shifts after sham/noise exposure. Click and pure tone (8, 16, and 32 kHz) ABR measurements were performed to assess normal hearing thresholds before beginning behavioral assessments and were performed again after sham/noise exposure to assess hearing loss induced by sham/noise exposure. Absolute threshold shifts (dB SPL) were determined from the absolute differences in thresholds measured after sham/noise exposure and are plotted for the two conditions (sham exposure, white bars; noised, gray bars) for the four stimuli (click, 8 kHz, 16 kHz and 32 kHz). Noise exposed mice showed significantly greater threshold shifts at 16 and 32 kHz compared to sham exposed mice (Mann Whitney rank comparison test). Values and original data are provided in Table 1 and S1 Table.

Click and pure tone (8, 16, and 32 kHz) ABR measurements were performed (as described in the Methods) to assess normal hearing thresholds before beginning behavioral assessments. ABR measurements were performed again after sham/noise exposure to assess hearing loss induced by noise exposure. Threshold shifts (dB SPL) were determined from the differences in absolute thresholds measured after sham/noise exposure and before (Fig 2). All mice included in the study had normal baseline hearing in both ears [19]. As expected, when comparing within click or test frequencies, sham exposure caused minimal (non-significant) changes in thresholds shifts (white bars in Fig 2, Table 1), whereas noise exposure caused significant increases in threshold shifts (gray bars in Fig 2, Table 1) at 16 and 32 kHz (Mann Whitney rank comparison test). This noise exposure paradigm and the resulting threshold shifts are consistent with the induction of tinnitus as described in other studies examining tinnitus in mouse models [16,17,20]. In our experiments, mice were noised under anesthesia with ketamine. To assess the effects of ketamine on noise-induced hearing loss, we examined threshold shifts following noise exposure with or without ketamine anesthesia in both C57BL/6J mice (used in thus study) and FVB/NJ mice (a strain that does not show age-related hearing loss). These experiments indicated that, in C57BL/6J mice, PTSs are not statistically different when noise exposure is done either awake (without ketamine) or under anesthesia (with ketamine). These findings are provided in S1 Text.

**Table 1:** Absolute threshold shifts (dB SPL) following sham/noise exposure.

Condition	Click	8 kHz	16 kHz	32 kHz
**Sham (n = 4)**	7.5 ± 2.5	7.5 ± 2.5	8.8 ± 3.8	11.3 ± 4.3
**Noised (n = 4)**	12.5 ± 5.2	10.0 ± 3.5	43.8 ± 3.1	40.0 ± 2.0

Because behavioral assays after sham/noise exposure require detection of white noise (80 dB SPL), ABR measurements were also used to test preservation of hearing after sham/noise exposure. In our experiments, noise exposed mice showed significantly greater threshold shifts at 16 and 32 kHz compared to sham exposed mice (gray bars, Fig 2, Mann Whitney rank comparison test, Table 1). Nevertheless, auditory thresholds were preserved in response to clicks and at 8 kHz, indicating they could hear the white noise stimulus as required by the behavioral assay. Together, these data show that the noise exposed mice examined in this study had hearing loss consistent with the induction of tinnitus in other paradigms but maintained sufficient hearing for subsequent behavioral assessment.

### Changes in movement in response to silent gaps before sham/noise exposure

**Fig 3:**
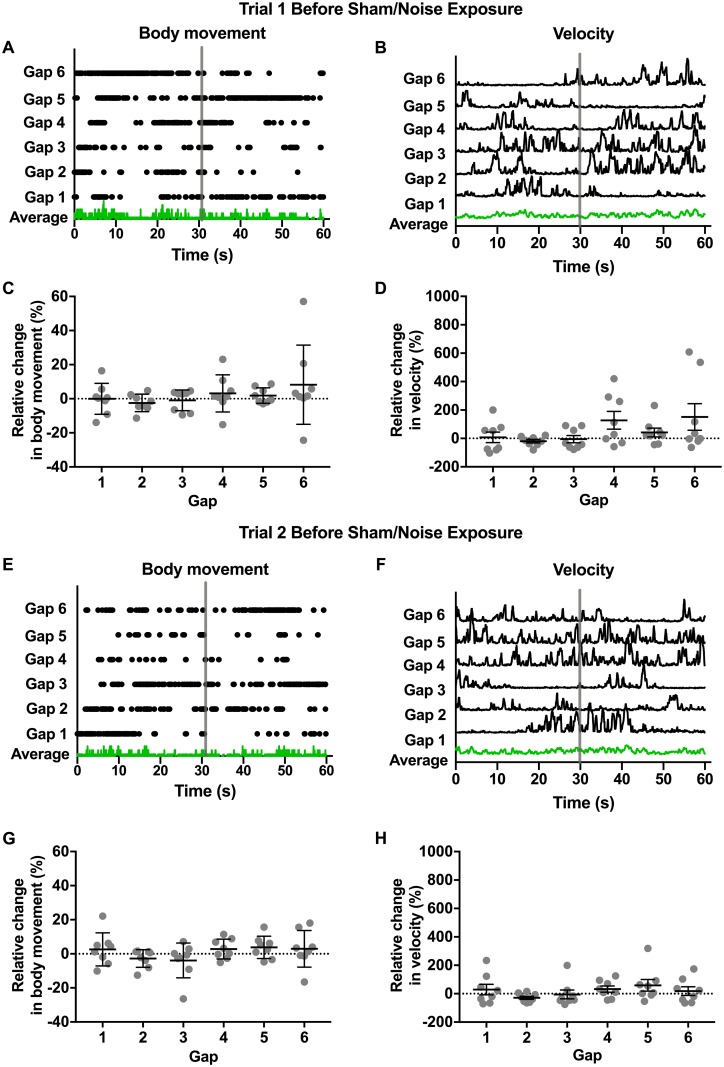
Changes in movement in response to silent gaps before sham/noise exposure. Changes in movement in response to silent gaps were measured in two trials before sham/noise exposure (trial 1 and 2). Each trial was 8.5 min in duration and contained 6 silent gaps of either 500 ms or 1 s in a background of white noise (80 dB SPL). Movement was quantified in two ways: 1) “body movement” or movement of the head, tail, and/or torso (center point) and 2) “velocity” or velocity measured at the torso (center point). **A, B, E, and F**. Body movement (A and E) and velocity (B and F) are plotted for an individual mouse for trial 1 (A and B) and trial 2 (E and F). To compare body movement and velocity across gaps from an individual trial, data are aligned across gaps (indicated by vertical gray bars) to show movement metrics for the 30 s preceding the onset of the silent gap, the 5 s containing the gap, and the subsequent 30 s. Because mice were generally very active, body movement plots (A and E) indicate periods of no movement during these time windows. Green traces indicate the average responses across the aligned time windows for the example mouse (A, B, E, and F). Across gaps, no consistent change in body movement or velocity are apparent. **C, D, G, and H**. To compare changes in movement during the gaps across mice for the first (C and D) and second (G and H) trials, relative changes in movement during the 5 s containing the gap and the preceding 30 s were calculated (as described in the Methods) for both body movement (C and G) and velocity (D and H). For both metrics, relative changes in movement were not observed in response to silent gaps (Table 2). Gray dots indicate individual values and black bars indicate mean and SEM. In all cases, no significant differences were observed across mice for individual gaps (Kruskal Wallis test with Dunn’s multiple comparisons). Values and original data are provided in Table 2 and S2 Table.

**Fig 4:**
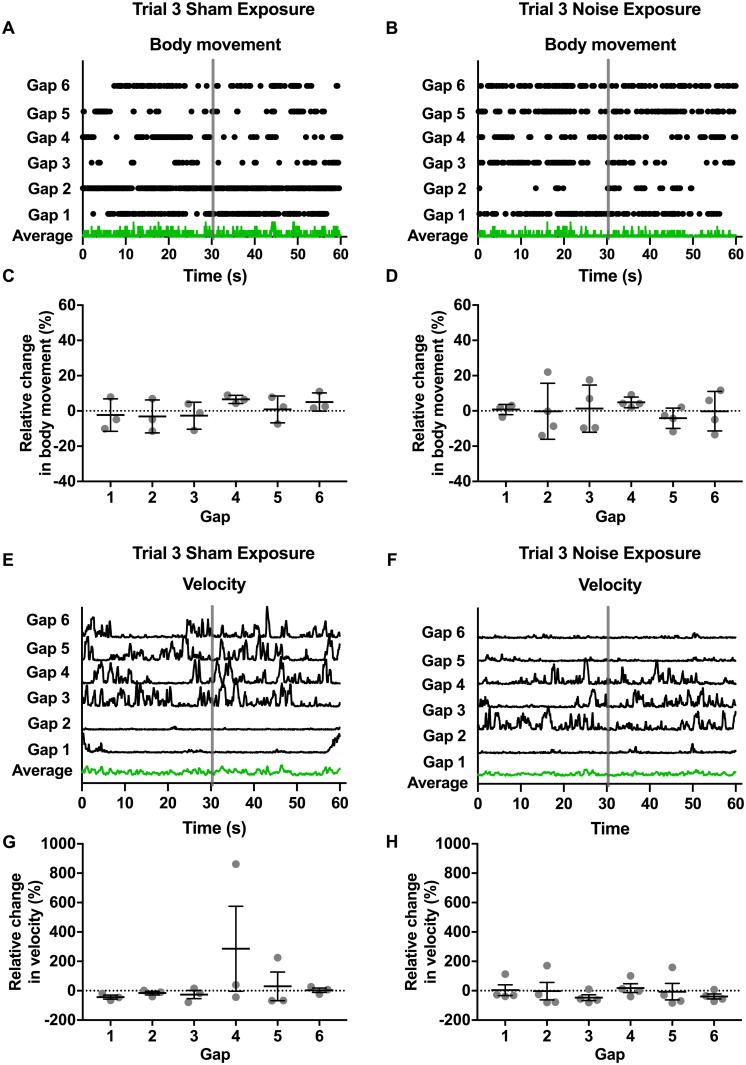
Changes in movement in response to silent gaps after sham/noise exposure. Changes in movement in response to silent gaps were measured again after sham/noise exposure (trial 3). These trials were also 8.5 min in duration and contained 6 silent gaps of either 500 ms or 1 s in a background of white noise (80 dB SPL). Movement was again quantified in two ways: 1) “body movement” or movement of the head, tail, and/or torso (center point) and 2) “velocity” or velocity measured at the torso (center point). **A-D**. Body movement (A and B) is plotted for a mouse that underwent sham (A) and noise (B) exposure. To compare body movement across gaps from these individual trials, data are aligned across gaps (indicated by vertical gray bars) to show body movement for the 30 s preceding the onset of the silent gap, the 5 s containing the gap, and the subsequent 30 s. Because mice were generally very active, body movement plots (A and B) indicate periods of no movement during these time windows. Green traces indicate the average responses across the aligned time windows for the sham (A) and noise (B) exposed mouse. To compare changes in body movement during the silent gaps across mice after sham (C) or noise (D) exposure, relative changes in movement during the 5 s containing the gap and the preceding 30 s were calculated (as described in the Methods) for body movement. Gray dots indicate individual values and black bars indicate mean and SEM. Relative changes in movement were not observed in response to silent gaps following either sham (C) or noise (D) exposure (Kruskal Wallis test with Dunn’s multiple comparisons test). **E-H**. Velocity (E and F) is plotted for a mouse that underwent sham (E) and noise (F) exposure. To compare velocity across gaps from these individual trials, data are aligned across gaps (indicated by vertical gray bars) to show body movement for the 30 s preceding the onset of the silent gap, the 5 s containing the gap, and the subsequent 30 s. Green traces indicate the average responses across the aligned time windows for the sham (E) and noise (F) exposed mouse. To compare changes in movement during the silent gaps across mice after sham (G) or noise (H) exposure, relative changes in movement during the 5 s containing the gap and the preceding 30 s were calculated (as described in the Methods) for velocity. Gray dots indicate individual values and black bars indicate mean and SEM. Relative changes in velocity were not observed in response to silent gaps following either sham (G) or noise (H) exposure (one-way analysis of variance). Values and original data are provided in Table 3 and S3 Table.

Based on previous observations in guinea pig [13], we expected normal hearing mice to show quantifiable changes in movement in response to silent gaps. We specifically expected decreases in movement (“freezing”) to indicate the perception of the sound gap. The perception of tinnitus was expected to “fill” this sound gap and cause a loss in this freezing response following noise exposure. We tested this prediction by monitoring movement over three trials, with two trials occurring before sham/noise exposure and one trial occurring after sham/noise exposure. Each trial was 8.5 min in duration and contained 6 silent gaps of either 500 ms or 1 s in a background of white noise (80 dB SPL). Movement was quantified in two ways: 1) body movement or movement of all three body markers, which included head, tail, and torso (center point) and 2) velocity (center point). These metrics are shown for an example mouse during the first trial (Fig 3A–3D). For these plots, the six gaps (indicated by the gray vertical bar) have been aligned to compare changes in movement in the 30 s just preceding the gap, the next 5 s containing the gap, and the subsequent 30 s. Because mice were generally very active, body movement plots indicate periods of no movement during these time windows. Green traces indicate the average response across aligned time windows for this example mouse during the first trial. Across gaps, no consistent changes in body movement or velocity are apparent. To compare changes in movement during the gaps across mice for the first trial, relative changes in movement during the 5 s containing the gap and the preceding 30 s were calculated (as described in the Methods) for both body movement (Fig 4C) and velocity (Fig 4D). For both metrics, relative changes in movement were not detected in response to silent gaps (Kruskal Wallis test with Dunn’s multiple comparisons test, Table 2).

**Table 2:** Relative changes in movement in response to silent gaps before sham/noise exposure (trials 1 and 2).

	Trial 1		Trial 2	
Gap	Relative change in body movement (%, n = 8)	Relative change in velocity (%, n = 8)	Relative change in body movement (%, n = 8)	Relative change in velocity (%, n = 8)
**1**	0.1 ± 3.2	7.5 ± 37.5	2.6 ± 3.4	29.1 ± 37.0
**2**	-2.4 ± 1.8	-19.3 ± 12.1	-2.8 ± 1.8	-28.0 ± 10.9
**3**	-0.9 ± 2.2	-4.8 ± 25.4	-3.9 ± 3.6	-5.1 ± 30.8
**4**	3.1 ± 3.8	127.7 ± 62.2	2.8 ± 2.1	32.3 ± 21.6
**5**	1.9 ± 1.6	41.9 ± 30.8	3.8 ± 2.3	59.1 ± 40.5
**6**	8.2 ± 8.2	151.2 ± 93.8	2.9 ± 3.8	18.2 ± 30.1

To verify the inter-test reliability of movement monitoring, we performed a second trial of movement monitoring (Fig 3E–3H). As described above, Fig 3E and 3F show body movement and velocity for the same mouse shown in Fig 3A and 3B over the six gaps. Green traces indicate the average response across aligned time windows for this example mouse during the second trial. As observed for the first trial, relative changes in movement were not detected in response to silent gaps for either movement metric in the second trial (Fig 3G and 3H, Table 2). Together, these experiments (Fig 3) indicate that there are consistent inter-trial measurements of relative movement (assessed as either body movement or velocity. However, in contrast to observations in guinea pig [13] and our first prediction, these results also indicate that mice do not show detectable “freezing” or other changes in movement in response to silent gaps as measured in our experiments.

### Changes in movement in response to silent gaps after sham/noise exposure

Although relative changes in movement during silent gaps were not detectable before noise exposure, we determined changes in relative movement in response to silent gaps after sham/noise exposure to examine whether changes then became evident that might indicate the perception of tinnitus (and thereby test our second prediction). We also wanted to examine whether noise exposure caused changes more generally in activity (and thereby test our third prediction). For these experiments mice were divided into two groups, one undergoing sham noise exposure and a second undergoing noise exposure (as described in the Methods) consistent with the induction of tinnitus [16,17,20]. These two groups of mice underwent a third trial of movement monitoring identical to that used for trials 1 and 2. Again body movement (Fig 4A–4D) and velocity (Fig 4E–4H) are plotted over the six gaps for a mouse that underwent sham exposure (Fig 4A and 4E) compared to a mouse that underwent noise exposure (Fig 4B and 4F). Green traces indicate the average response across aligned time windows for these example mice during the third trials. To examine changes in movement during the gaps across sham versus noise exposed mice, relative changes in movement for both body movement (Fig 4C and 4G) and velocity (Fig 4D and 4H) were calculated (as described in the Methods). Relative changes in movement (measured as either body movement or velocity) were not detected in response to silent gaps for either sham or noise exposed mice (Kruskal Wallis test with Dunn’s multiple comparisons test, Table 3).

**Table 3:** Relative changes in movement in response to silent gaps after sham/noise exposure (trial 3).

	Sham exposure		Noise exposure	
Gap	Relative change in body movement (%, n = 3*)	Relative change in velocity (%, n = 3*)	Relative change in body movement (%, n = 4)	Relative change in velocity (%, n = 4)
**1**	-2.3 ± 5.3	-43.23 ± 13.5	0.8 ± 1.5	3.7 ± 36.5
**2**	-3.1 ± 5.4	-15.9 ± 12.3	-0.2 ± 7.9	-2.8 ± 59.3
**3**	-2.7 ± 4.4	-26.0 ± 28.0	1.3 ± 6.7	-46.9 ± 19.2
**4**	6.6 ± 1.3	286.1 ± 289	4.9 ± 1.5	18.1 ± 29.9
**5**	0.9 ± 4.4	30.1 ± 97.4	-4.1 ± 2.9	-6.2 ± 56.1
**6**	5.1 ± 3.0	3.6 ± 14.7	-0.2 ± 5.6	-38.9 ± 17.3

*One mouse failed to move at all after sham exposure and was not included in these calculations.

**Fig 5:**
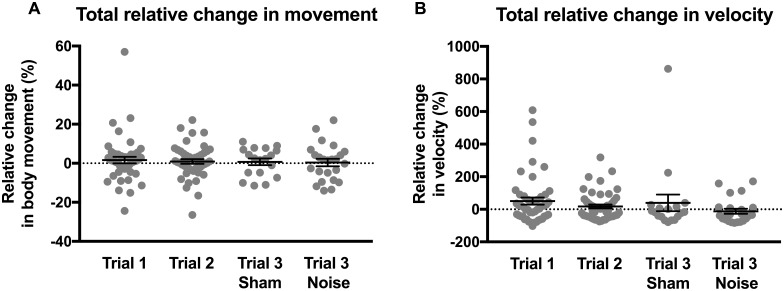
Comparison of the changes in movement in response to silent gaps across trials (before and after sham/noise exposure). Because significant differences in relative movement were not observed between mice and/or gaps in a given trial, these data were pooled to compare differences in relative movement across the four trial conditions: trial 1 (before sham/noise exposure), trial 2 (before sham/noise exposure), trial 3 (after sham exposure), and trial 3 (after noise exposure) for body movement (A) and velocity (B). Gray dots indicate individual values and black bars indicate mean and SEM. No differences in relative changes in movement were observed in response to silent gaps between the four trial conditions when comparing either movement metric (Kruskal Wallis test with Dunn’s multiple comparisons test). Values and original data are provided in Table 4 and S4 Table.

Because significant differences in relative movement were not detected between mice and/or gaps in a given trial, these data were pooled to compare differences in relative movement across the four trial conditions (Fig 5): trial 1 (before sham/noise exposure), trial 2 (before sham/noise exposure), trial 3 (after sham exposure), and trial 3 (after noise exposure). No differences in relative changes in movement were observed in response to silent gaps between the four trial conditions when comparing either movement metric, body movement (Fig 5A) or velocity (Fig 5B, Kruskal Wallis test with Dunn’s multiple comparisons test, Table 4). These results reiterate that, in contrast to observations in guinea pig [13], normally hearing mice, which include the mice in trial 1, trial 2, and trial 3 (after sham exposure), do not show detectable changes in movement in response to silent gaps. Moreover, these data show that mice exposed to noise that causes hearing loss and presumably tinnitus, which include the mice in trial 3 (after noise exposure), also show no detectable changes in movement in response to silent gaps.

**Table 4:** Relative changes in movement across the four trial conditions.

Trial Condition	Relative change in body movement (%)	Relative change in velocity (%)
**Trial 1 (before sham/noise exposure, n = 8 for 6 gaps)**	1.6 ± 1.7	50.7 ± 22.0
**Trial 2 (before sham/noise exposure, n = 8 for 6 gaps)**	0.9 ± 1.2	17.6 ± 12.3
Trial 3 (after sham exposure, n = 3 for 6 gaps*)	0.7 ± 1.7	39.1 ± 51.0
**Trial 3 (after noise exposure, n = 4 for 6 gaps)**	0.4 ± 1.9	-12.2 ± 15.2

*One mouse failed to move at all after sham exposure and was not included in these calculations.

### Changes in total activity after noise exposure

**Fig 6:**
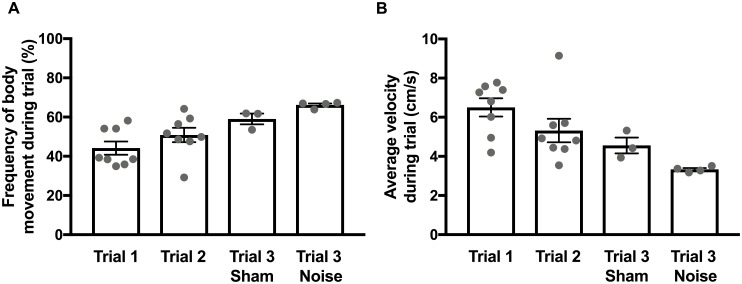
Comparison of the changes in total activity across trials (before and after sham/noise exposure). To examine changes in total activity across trials, the average activity across the entire trial (8.5 min) was calculated for the frequency of body movement (A) and velocity (B) for each mouse. Gray dots indicate individual values and black bars indicate mean and SEM. No significant differences (Friedman test with Dunn’s multiple comparison test) in total activity across trials were observed when examining the frequency of body movement (A) or average velocity (B) indicated no significant difference in total activity from the first trial to the last (third) trial. Values and original data are provided in Table 5 and S5 Table.

Lastly, to examine changes in total activity after noise exposure, we compared total activity across mice over the entire duration of the four trial conditions (Table 5; Fig 6): trial 1 (before sham/noise exposure), trial 2 (before sham/noise exposure), trial 3 (after sham exposure), and trial 3 (after noise exposure). This comparison was motivated by the previous observation that overall movement was increased in noise-exposed guinea pigs predicted to have tinnitus [13]. This observation prompted our third prediction that noise exposed mice would show greater total activity compared to activity before sham/noise exposure and after sham exposure. In contrast to expectation, the total activity as measured by either frequency of body movement (Fig 6A) or average velocity (Fig 6B) showed no significant differences across trials (Friedman test with Dunn’s multiple comparison test). These data indicate that noise exposure causing hearing loss and expected to induce tinnitus does not cause an increase in movement in mice as observed in guinea pigs.

**Table 5:** Total activity across the four trial conditions.

Trial Condition	Frequency of body movement (%)	Average velocity (cm/s)
**Trial 1 (before noise exposure, n = 8)**	44.2 ± 3.4	6.5 ± 0.5
**Trial 2 (before noise exposure, n = 8)**	50.8 ± 3.7	5.3 ± 0.6
Trial 3 (after sham exposure, n = 3*)	59.0 ± 2.8	4.6 ± 0.4
**Trial 3 (after noise exposure, n = 4)**	66.2 ± 0.8	3.3 ± 0.1

*One mouse failed to move at all after sham exposure and was not included in these calculations.

## Discussion

For a variety of reasons paradigms for identifying the perception of tinnitus in mice have been difficult to establish. As a result, mice have been underutilized to investigate the cellular, molecular, and genetic mechanisms underlying tinnitus. In this work, we investigated the efficacy of changes in spontaneous (unconditioned) movement in response to silent gaps to indicate the perception of tinnitus in mice. This work was based on previous observations in guinea pigs that reported changes in movement during silent gaps that were unique to a subgroup of guinea pigs presumably with tinnitus [13]. Specifically, guinea pigs presumably with tinnitus failed to “freeze” in response to silent gaps in sound, likely because the tinnitus filled the silent gap. The overall hope was to develop a rapid and reliable assay for identifying the perception of tinnitus in mice. The ability to assay an unconditioned rather than learned response to detect would enormously accelerate the use of mouse models in tinnitus research. In adapting this assay from guinea pig to mice, several differences in their auditory and behavioral response were considered, most notably differences in their frequency ranges of hearing and their overall level of activities.

Based on this earlier work in guinea pig, we had three predictions in mice. First, we expected that normally hearing mice would show detectable and reliable changes in movement (likely “freezing”) in response to silent gaps. Second, we expected that mice would show changes in movement (likely lack of “freezing”) in response to silent gaps after noise exposure expected to cause tinnitus compared to normally hearing and sham exposed mice. Third, based on additional observations in guinea pigs, we expected mice to have increased overall movement after noise exposure expected to cause tinnitus. As our results show, none of these predictions was supported. We observed no detectable changes in movement in normally hearing mice or in mice following noise exposure expected to produce tinnitus. We also found that noise exposure that causes hearing loss and is expected to induce tinnitus caused no significant change in overall movement.

These results indicate that changes in spontaneous movement during silent gaps, as presented in this study, are not effective for the detection of tinnitus perception in mice. Specifically, the variability in spontaneous movement both before and after sham/noise exposure was substantially greater in mice than previously reported in guinea pigs [13]. The larger variability in effect size indicates that a larger sample size than used in this study is necessary to detect significant differences in spontaneous movement using this assay. Using the observed effect size in mice, we performed a power analysis to determine the number of mice required to to detect statistically significant differences in spontaneous movement as described in this assay. The variability in effect size is, in fact, so great that an experimentally and ethically unreasonable number of animals (estimated at n = 7,600) would be required. Thus, this assay is not sufficiently robust to identify changes in movement that might indicate the perception of tinnitus in mice. The substantially reduced effect size (changes in spontaneous movement in response to silent gaps) observed in mice compared to guinea pig [13], may suggest that the mouse strain used in this study (and likely other strains as well) simply do not to attend to the background noise and/or silent gap. Likewise, gap detection may be preserved after noise exposure expected to cause tinnitus. Indeed, gap detection deficits in mice with cochlear neuropathy are limited [21] and gap detection is normal in tinnitus patients [22,23]. These observations may suggest that gap detection is simply not a robust enough indicator of tinnitus perception in mice.

Despite the lack of robustness of the assay reported here, other unconditioned and also conditioned behaviors should be examined in mice to test their utility to detect changes that indicate the perception of tinnitus. Moreover, other indicators of tinnitus perception, for example detection of more complex auditory stimuli beyond gap detection, should be considered and examined.

## Supporting information

S1 Table Original ABR threshold shift data. (XLSX)

S2 Table Original trial 1 and trial 2 movement data. (XLSX)

S3 Table Original trial 3 movement data. (XLSX)

S4 Table Original cross-trial comparison data. (XLSX)

S5 Table Original total activity data. (XLSX)

S1 Text Effect of ketamine anesthesia on temporary and permanent threshold shifts and ribbon synapse number after noising in C57BL/6J and FVB/NJ mice. (PDF)
