# Supplementary material for: Changes in spontaneous movement in response to silent gaps are not robust enough to indicate the perception of tinnitus in mice
Source: PLoS One. 2018 Aug 29;13(8):e0202882. doi: 10.1371/journal.pone.0202882 (PMC6114799; doi:10.1371/journal.pone.0202882)
Supplement: S1 Text — (PDF) [file pone.0202882.s006.pdf]

Supplementary File S1

Introduction: In our experiments, mice were noised under anaesthesia with ketamine. To assess the effects of ketamine on noise-induced hearing loss, we examined threshold shifts following noise exposure with or without ketamine anaesthesia in both C57BL/6J mice (used in thus study) and also FVB/NJ mice (a strain that does not show age-related hearing loss). We additionally examined ribbon synapse number (loss) associated with permanent threshold shifts (7 days after noising) in both strains.

Methods: ABRs were done as described in the Methods and were performed 7 days before noise exposure and 1 day and 7 days after noise exposure to assess temporary and permanent threshold shifts (TTS and PTS), respectively. Noising was done also as described in the Methods except that the noise stimulus was an octave band of noise (8-16 kHz) at 98 dB SPL for 2 h and was done on either anaesthetized (with ketamine) or awake C57BL6/J or FVB/NJ mice aged 6 weeks. Ribbons were identified by immunofluorescence, confocal microscopy and quantification as described previously (Braude et al., 2015). A total of 6 C57BL/6J mice (3 with ketamine and 3 without ketamine) and 6 FVB/NJ mice (also 3 with ketamine and 3 without ketamine) were investigated. Baseline (without noise exposure) ribbon synapse counts came from previous experiments (n = 8 for C57BL/6J mice and n = 3-4 for FVB/NJ mice). Significant differences within test frequencies were assessed using a Mann Whitney rank comparison test.

Results: There are three important findings from these experiments. First, C57BL/6J mice showed greater noise-induced TTSs (A, B) and PTSs (C, D) compared to FVB/NJ mice (regardless of ketamine application). Second, within test frequencies there were no significant differences in TTSs (A, B) or PTSs (C, D) with or without ketamine application in either C57BL/6J or FVB/NJ mice. Third, within test frequencies there were no significant differences in ribbon synapse number (loss) associated with PTSs with or without ketamine application in either C57BL/6J (E) or FVB/NJ (F) mice.

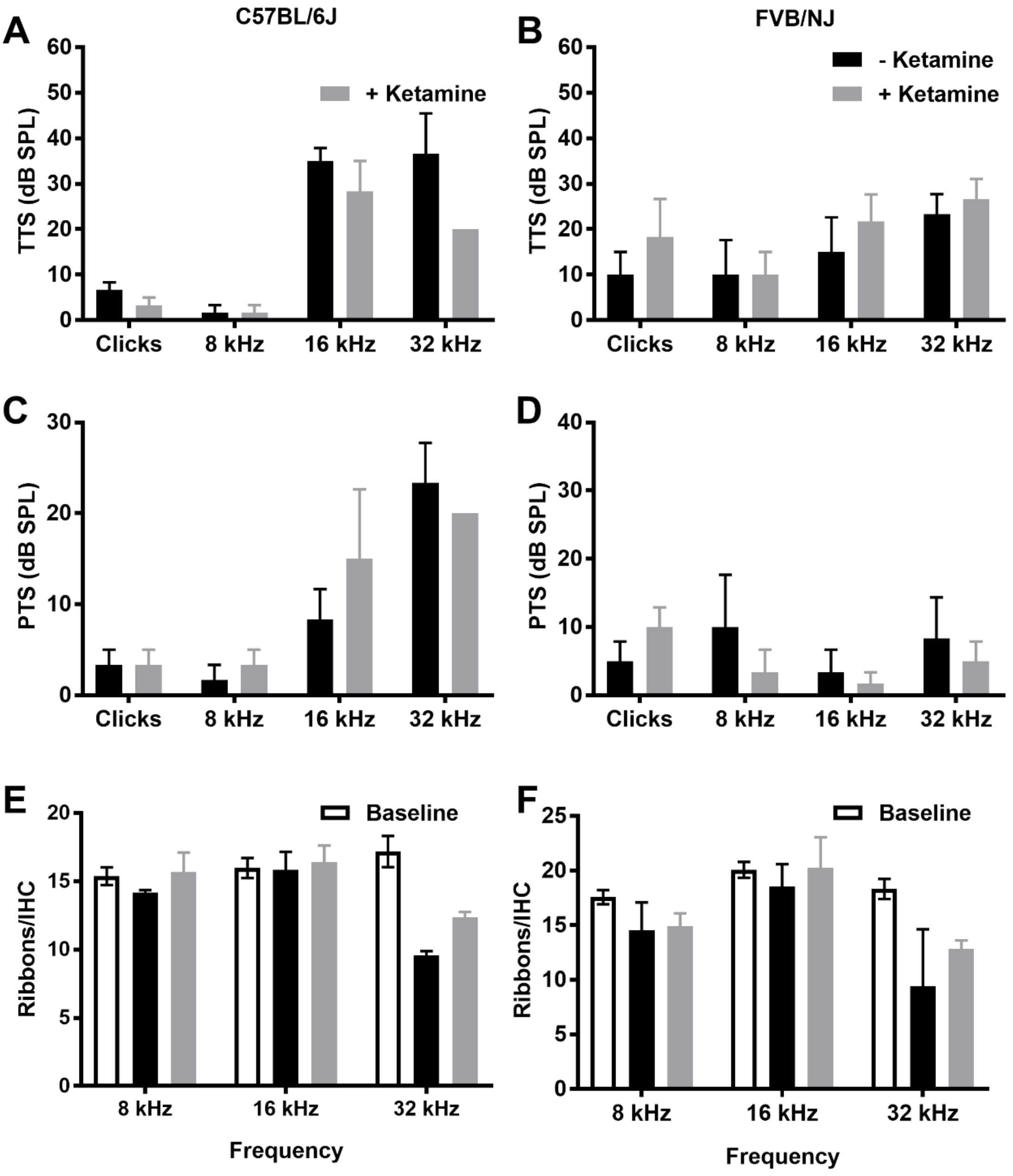

Conclusions: Under these conditions ketamine application had no effect on the magnitude of either TTSs or PTSs. We expect that the lack of effect of ketamine application on noise-induced hearing loss is consistent across noise exposure regimes but the effect of noise exposure regimes could be investigated further. These experiments also suggest that there are strain-dependent effects on recovery from noise-induced threshold shifts and ribbon synapse loss follow noise exposure that could be investigated further.
